# Nicotine effect on bone remodeling during orthodontic tooth movement: Histological study in rats

**DOI:** 10.1590/2176-9451.19.2.096-107.oar

**Published:** 2014

**Authors:** Ricardo Lima Shintcovsk, Luégya Knop, Orlando Motohiro Tanaka, Hiroshi Maruo

**Affiliations:** 1 Professor, Brazilian Dental Association.; 2 Full professor, Catholic Univeristy of Paraná (PUC-PR).; 3 PhD in Orthodontics, State Univeristy of Campinas (UNICAMP).

**Keywords:** Tooth movement, Bone resorption, Bone formation, Blood vessels, Nicotine

## Abstract

**Introduction:**

Nicotine is harmful to angiogenesis, osteogenesis and synthesis of collagen.

**Objective:**

The aim of this study was to investigate the effect of nicotine on bone remodeling during orthodontic movement in rats.

**Methods:**

Eighty male Wistar rats were randomly divided into three groups: Group C (control), group CM (with orthodontic movement) and group NM (nicotine with orthodontic movement) groups. The animals comprising groups C and CM received 0.9% saline solution while group NM received nicotine solution (2 mg/kg). A nickel-titanium closed-coil spring was used to induce tooth movement. The animals were euthanized and tissue specimens were processed histologically. We quantified blood vessels, Howship's lacunae and osteoclast-like cells present in the tension and compression areas of periodontal ligaments. The extent of bone formation was evaluated under polarized light to determine the percentage of immature/mature collagen.

**Results:**

We observed lower blood vessel densities in the NM group in comparison to the CM group, three (p < 0.001) and seven (p < 0.05) days after force application. Osteoclast-like cells and Howship's lacunae in the NM group presented lower levels of expression in comparison to the CM group, with significant differences on day 7 (p < 0.05 for both variables) and day 14 (p < 0.05 for osteoclast-like cells and p < 0.01 for Howship's lacunae). The percentage of immature collagen increased in the NM group in comparison to the CM group with a statistically significant difference on day 3 (p < 0.05), day 7 (p < 0.001), day 14 (p < 0.001) and day 21 (p < 0.001).

**Conclusions:**

Nicotine affects bone remodeling during orthodontic movement, reducing angiogenesis, osteoclast-like cells and Howship's lacunae, thereby delaying the collagen maturation process in developed bone matrix.

## Abstract (pt)

**Introdução:**

a nicotina apresenta efeito prejudicial sobre a angiogênese, osteogênese e síntese de colágeno.

**Objetivo:**

investigar a ação da nicotina sobre a remodelação óssea durante o movimento dentário induzido em ratos.

**Métodos:**

oitenta ratos machos Wistar foram divididos em três grupos: grupo C (sem indução de movimento dentário e sem a ação da nicotina - controle); grupo CM (indução de movimento dentário) e grupo NM (indução de movimento dentário associado à ação da nicotina). Os animais dos grupos C e CM receberam solução salina a 0,9% e os animais do grupo NM receberam nicotina (solução PA a 98% diluída em solução salina a 0,9% estéril) por via subcutânea (2mg/kg). Após a eutanásia dos animais, com 3, 7, 14 e 21 dias de uso da mola ortodôntica, os espécimes teciduais foram processados histologicamente e quantificou-se o número de vasos sanguíneos, lacunas de Howship e células osteoclásticas nos lados de tração e compressão do ligamento periodontal. A neoformação óssea foi avaliada por meio de luz polarizada, para determinar a porcentagem de colágeno maduro e imaturo.

**Resultados:**

observou-se que a quantidade de vasos sanguíneos diminuiu no grupo NM, quando comparado ao grupo CM, nos períodos de três (p < 0,001) e sete (p < 0,05) dias. Quanto às células osteoclásticas e lacunas de Howship, o grupo NM apresentou menores níveis de expressão em relação ao grupo CM, com diferença estatisticamente significativa nos períodos de 7 e 14 dias. A porcentagem de colágeno imaturo apresentou-se aumentada no grupo NM, quando comparado ao grupo CM, em todos os períodos analisados, com diferença estatisticamente significativa.

**Conclusão:**

a nicotina interferiu no processo de remodelação óssea durante o movimento dentário induzido, reduzindo a angiogênese, células osteoclásticas e lacunas de Howship, e atrasando a maturação do colágeno da matriz óssea neoformada.

## INTRODUCTION

Smoking and other forms of tobacco use are major risk factors for cardiovascular disease. The effect of cigarette smoking on cardiovascular health is evident even at the lowest levels of exposure.^1^ Nicotine (*Nicotiana tabacum*) is the most pharmacologically active component present in tobacco and directly or indirectly affects cellular metabolism, reducing angiogenesis;^2^ osteogenesis;^3,4^ fibroblasts proliferation and adhesion as well as collagen synthesis.^5^

According to Hollinger et al^6^ and Feitelson et al,^7^ nicotine exhibits broad pharmacological action, of which the biggest effect is vasoconstriction. Zhu and Parmley^8^ reported that the mechanisms that produce this vasoconstriction are associated with local or systemic catecholamine release, sympathetic neural stimulation, and endothelial dysfunction. Nicotine induces norepinephrine release from postganglionic sympathetic nerves, innervating blood vessels through a direct action on the nerve terminals.^9^ Consequently, systemic and local actions occur, which can influence the biological processes that require higher metabolic activity.^2,3^

The early phase of orthodontic tooth movement always involves an acute inflammatory response, characterized by periodontal vasodilatation and migration of leucocytes out of the capillaries. These migratory cells produce various cytokines, the local biomechanical signal molecules that interact with the entire population of native paradental cells. Cytokines evoke the synthesis and secretion of numerous mediators by target cells, including growth factors, prostaglandins and other cytokines. Subsequent biological events occur and result in bone remodeling to accommodate movement of the tooth.^10,11^

Bone resorption and bone formation are parts of the remodeling process during orthodontic tooth movement. Bone is deposited on the alveolar wall on the tension side of the tooth with both heavy and light forces, and newly formed bone spicules follow the orientation of the periodontal fiber bundles. On the pressure side, with light forces, alveolar bone is directly resorbed by numerous osteoclasts in Howship's lacunae.^11^

Although there are more than 1.3 billion of smokers in the world^12^ and the acceptance of the fact that nicotine acts on cellular and tissue metabolism is widespread, there are no reports in the literature demonstrating the action of nicotine on orthodontic movement.

Thus, the aim of this study was to investigate the effect of nicotine on bone remodeling during orthodontic movement induced in rats.

## MATERIAL AND METHODS

The present study was approved by the Committee on Animal Research and Ethics of the Catholic University of Paraná (PUCPR), under protocol number 199/07.

A total of 80 male Wistar albino rats, 12 weeks old and weighing from 250 to 300 g, were used in this study. Animals were kept in polycarbonate boxes at temperatures ranging between 19ºC and 22ºC, with a standard 12-hour light-dark cycle. They were fed a diet of finely ground laboratory food *ad libitum* to minimize any discomfort to the animal following orthodontic appliance placement. The animals weight decreased during the experiment, but without significantly statistic differences (p > 0.05).

The rats were divided into three groups: Group C (control), group CM (with orthodontic movement) and group NM (nicotine with orthodontic movement). The study focused on the mesio-buccal roots of maxillary first molars. The right hemi-maxillae comprised groups CM and NM (40 rats in each) while the left hemi-maxillae comprised group C. The use of contralateral molars as control was according to Ong et al,^13^ Kalia et al,^14^ Bletsa et al,^15^ and Ren et al.^16^

The animals of groups C and CM received 0.9% saline solution at 0.5 ml/kg every 24 hours so as to simulate stress. Group NM received daily doses of 2 mg/kg nicotine solution (98% PA solution diluted in 0.9% saline solution), subcutaneously. The dosage was based on the study conducted by Chen et al.^17^ The applications began one day before orthodontic appliance placement and were reapplied once a day during the experimental periods of three, seven, 14 and 21 days.

**Figure 1:**
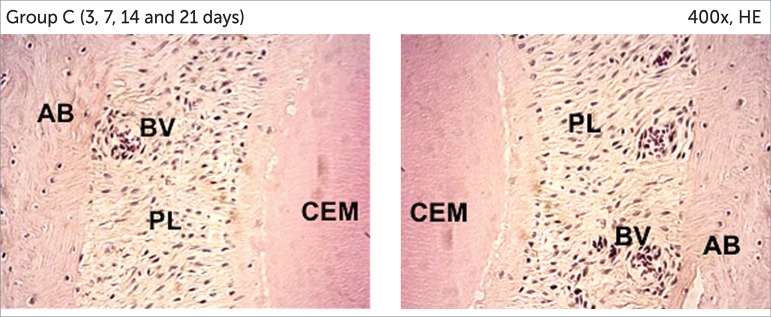
First molar mesiobuccal roots photomicrographs from group C (AB= alveolar bone; CEM= cementum; PL= periodontal ligament; BV= blood vessels).

Orthodontic movement was induced by nickel-titanium closed-coil springs (G&H^®^ Wire Company REF CCOF9XL Lote 103946 Hanover, Germany) that applied a reciprocal force between the maxillary right first molar and central incisors (Fig 1) of 30 g/f magnitude, measured by a Dynamometer gauge (Dentaurum model stress and tension gauge, 25-250 g/f). The coil spring was inserted while the animal was sedated with intramuscular injection of 1.8 mg/kg ketamine (Vetanarcol^®^, Konig, Avellaneda, Argentina) and 1.1 mg/kg xylazine (Rompun^®^, Bayer, Lote 00404, São Paulo, Brazil). During the experiment, all coil springs were evaluated and should any of them fail, the animal would be replaced by another one.

Animals were euthanized three, seven, 14 and 21 days after the orthodontic appliance was placed, with ketamine (5.4 mg/kg) and xylazine (3.3 mg/kg), via intraperitoneal injection. After euthanasia, the maxillae were immediately removed and fixed in 10% neutral formalin for 72 hours. The resulting blocks were decalcified for approximately 12 weeks in a 4.13% EDTA aqueous solution. 5-μm-thick transversal cuts were obtained and stained by means of conventional methods. From each maxillary block, 16 cuts were obtained from the alveolar crest up to the apices; 12 of which were stained with hematoxylin-eosin (HE) and four with picrosirius.

The histological study was performed by one operator, blinded to treatment allocation. Osteoclast-like cells, active Howship's lacunae and blood vessels were quantified under 400 x magnification using a light microscope. The histologic criterion used to identify the osteoclast-like cells was the presence of multinuclear and eosinophilic cells on the bone surface.^18^

Histological sections stained by the picrosirius method were viewed under 100 x magnification with a polarized light microscope. This method allows an indirect evaluation of the stage of bone matrix organization based on birefringence of the collagen fiber bundles.^19^ The analysis was performed by 4.5 Image Pro-Plus^®^ software (Media Cybernetics, Silver Spring, MD, USA) which calculated the percentages of immature and mature collagen present on bone matrix proximal to the tension area.

The mean values obtained were statistically analyzed by means of the SPSS 15.0 (SPSS Inc, Chicago, IL, USA) and Statistica 8.0 (StatSoft, Inc, Tulsa, OK, USA) software. The Kolmogorov-Smirnov test and Levene test were used to evaluate normality for each treatment and the homogeneity of variance between treatments. When the tests indicated non-normal distribution and heterogeneity of variance between treatments, we applied the non-parametric test and the non-parametric Kruskal-Wallis test for multiple comparisons. When the tests indicated normal distribution and homogeneity of variance, we used ANOVA and the Student's t-test for independent samples. The level of significance was p < 0.05 when comparing the averages between treatments and groups.

## RESULTS

### Histology

The histological study demonstrated characteristic structural aspects among different groups throughout the experimental period.

#### Group C

The periodontal ligament showed moderate vascularization, with uniform width and irregular shape. The collagen fibers ran parallel and inserted perpendicular to the cementum and bone surface. On the bone surface, rare Howship's lacunae associated with osteoclast-like cells were observed (Fig 1).

#### Group CM

**Figure 2:**
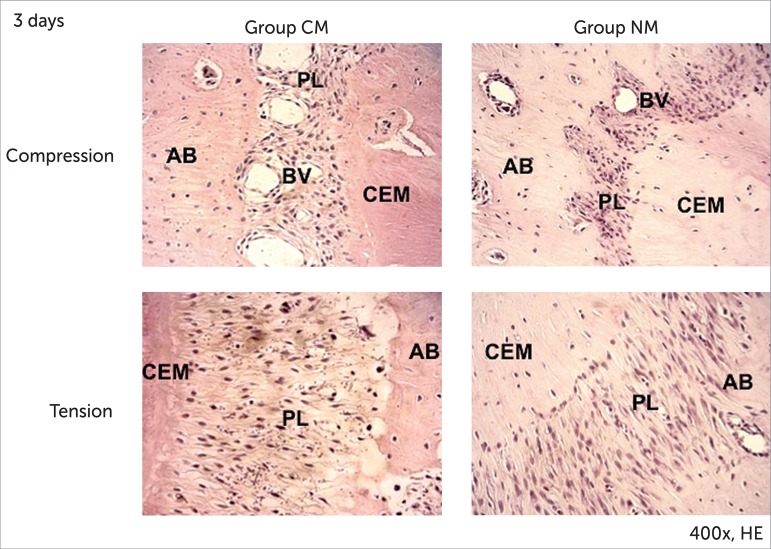
First molar mesiobuccal roots photomicrographs from groups CM and NM. Compression and Tension areas 3 days (HE - original magnification x400). AB, alveolar bone; CEM, cementum; PL, periodontal ligament; BV, blood vessels.

In the sections of these samples, on day three, the collagen fibers were elongated on the tension areas, compressed and completely disorganized on the compression areas. We also observed angiogenesis with congested vessels. The compression area showed irregular alveolar bone due to a small population of osteoclast-like cells localized on Howship's lacunae. Hyalinized areas were not present (Fig 2). The bone matrix under polarized light demonstrated a predominance of greenish, immature fibers with irregular birefringence and few yellowish-orange fibers (Fig 6).

**Figure 3:**
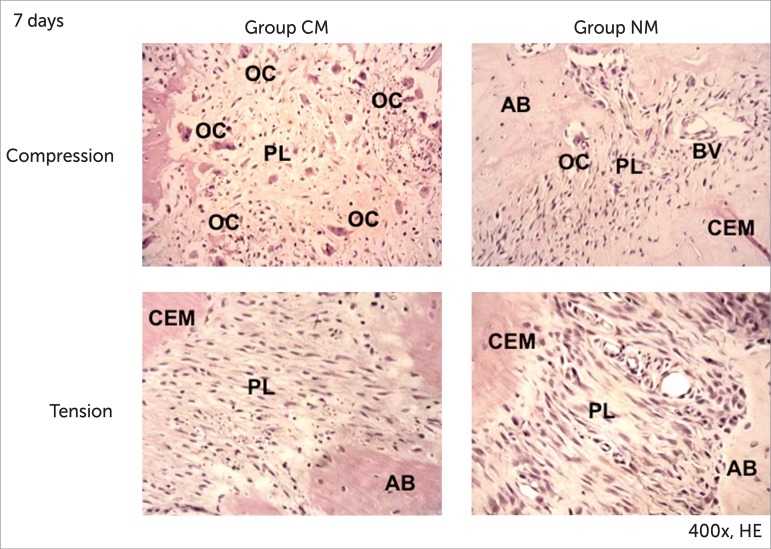
First molar mesiobuccal roots photomicrographs from groups CM and NM. Compression and Tension areas 7 days (HE - original magnification x400). AB, alveolar bone; CEM, cementum; PL, periodontal ligament; BV, blood vessels; OC, osteoclast-like cells.

**Figure 4:**
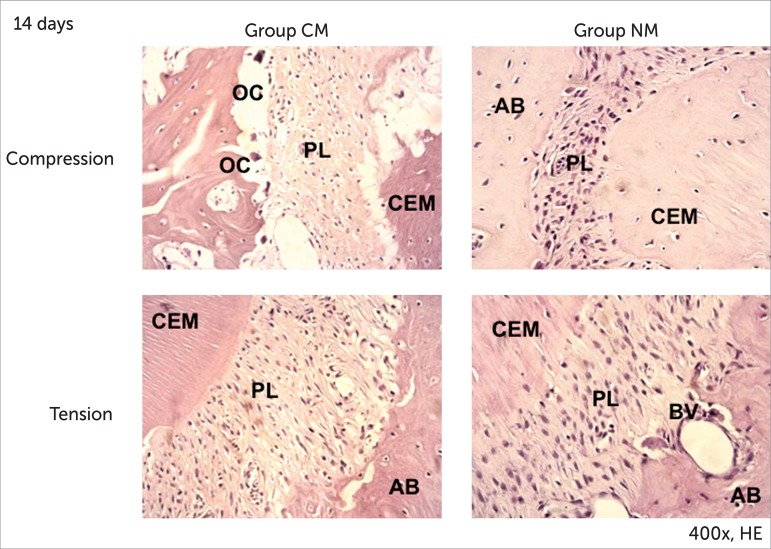
First molar mesiobuccal roots photomicrographs from groups CM and NM. Compression and Tension areas 14 days (HE - original magnification x400). AB, alveolar bone; CEM, cementum; PL, periodontal ligament; BV, blood vessels; OC, osteoclast-like cells.

On day seven, the compression area showed active resorption, with accumulation of osteoclast-like cells associated with numerous Howship's lacunae. In the tension area, the fibers were elongated and better organized (Fig 3). We observed deposition of thicker and yellowish-orange collagen fibers, forming more compact areas (Fig 6). On day 14, in the compression area, osteoclast-like cells and Howship's lacunae were limited in number (Fig 4). In the tension area, the periodontal ligament displayed aspects of normality (Fig 7). The developed bone matrix was completely filled by red and thick collagen fibers.

**Figure 5:**
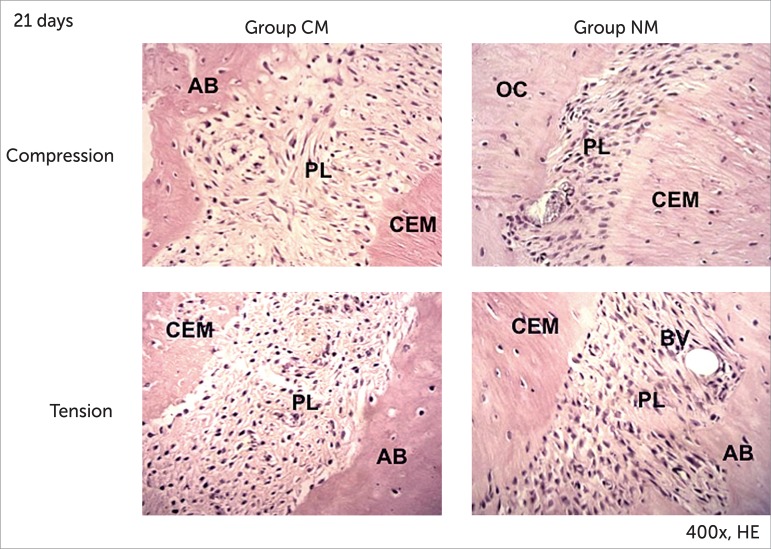
First molar mesiobuccal roots photomicrographs from groups CM and NM. Compression and Tension areas: 21 days (HE - original magnification x400). AB, alveolar bone; CEM, cementum; PL, periodontal ligament; BV, blood vessels.

On day 21, we observed that osteoclast-like cells and Howship's lacunae returned to normal basal expression levels in the compression area. In the tension area, the collagen fibers were more organized, parallel, and inserted perpendicular to the alveolar bone and cementum (Fig 5). The developed bone matrix was filled by mature collagen (Fig 7).

#### Group NM

**Figure 6:**
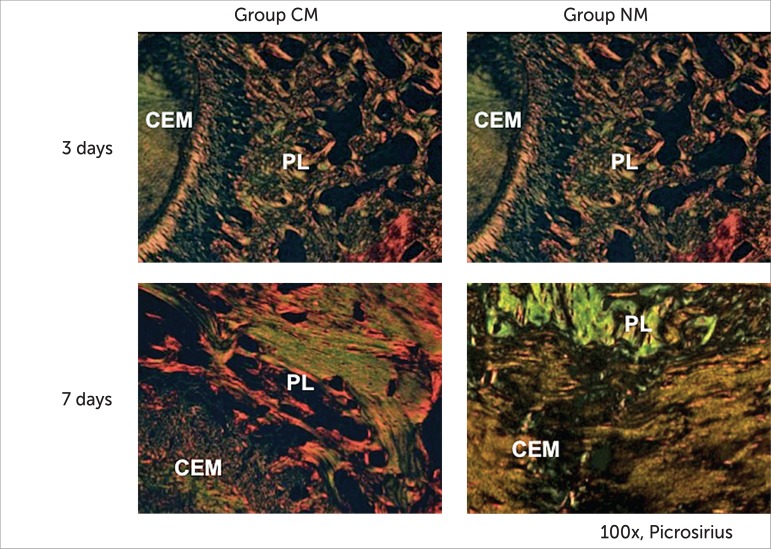
First molar mesiobuccal roots photomicrographs groups CM and NM. Tension areas: 3 and 7 days (picrosirius - original magnification x100). CEM, cementum; PL, periodontal ligament.

On day three, we observed the compression and tension areas. The periodontal ligament on the compression side demonstrated nearly none osteoclast-like cells or Howship's lacunae. No hyalinized zones were present. In the tension area, the periodontal ligament fibers were elongated, oriented parallel to the root. Some blood vessels were visualized (Fig 2). Under polarized light, we observed a predominance of greenish and thinner fibers in the bone matrix (Fig 6). On day seven, the compression area showed some osteoclast-like cells and Howship's lacunae. No hyalinized zones were present.

In the tension area, the fibers were elongated and fewer blood vessels were visualized (Fig 3). We observed a predominance of greenish collagenous fibers, extending in several directions, with irregular birefringence (Fig 6).

**Figure 7:**
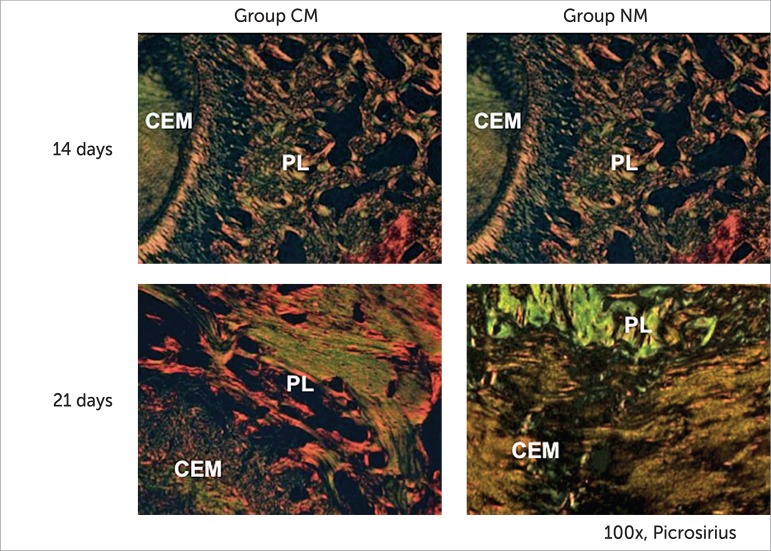
First molar mesiobuccal roots photomicrographs groups CM and NM. Tension areas: 14 and 21 days (picrosirius - original magnification x100). CEM, cementum; PL, periodontal ligament.

On day 14, in the compression area, the periodontal ligament fibers were disorganized and irregular; few Howship's lacunae and osteoclast-like cells were present. In the tension area, the periodontal ligament fibers remained elongated and few blood vessels were visualized (Fig 4). The bone matrix presented a large number of greenish fibers (Fig 7). On day 21, in the compression and tension areas, the periodontal ligament fibers were disorganized. Few osteoclast-like cells, Howship's lacunae and blood vessels were present (Fig 5). It was possible to verify, in the bone matrix, a large number of greenish fibers, thick and parallel to each other, as well as a few thinner red fibers (Fig 7).

### Quantitative analysis

All means, standards-deviation (SD) and p-values obtained are shown in Tables 1 and 2.

**Table 1:** Means, standard deviations and p values, according to groups and days.

Variable	Day	C	CM	NM	Inter-group differences
		Mean ± SD	Mean ± SD	Mean ± SD	(p)
Osteoclasts-like cells	3	0.6 ± 0.84	6.3 ± 1.34	3.6 ± 1.35	***C x CM. *C x NM
	7	0.5 ± 0.71	16.9 ± 3.35	2.2 ± 1.14	***C x CM. *CM x NM
	14	0.7 ± 0.95	3.3 ± 1.06	1.2 ± 1.62	**C x CM. *CM x NM
	21	0.8 ± 0.92	1.4 ± 1.26	1.2 ± 1.32	N.S.
Howship lacunae	3	0.4 ± 0.52	6.5 ± 1.96	3.1 ± 1.79	***C x CM. *C x NM
	7	0.3 ± 0.48	17.8 ± 2.57	1.9 ± 1.52	***C x CM. *CM x NM
	14	0.5 ± 0.71	3.9 ± 1.2	0.6 ± 0.97	**C x CM. **CM x NM
	21	0.7 ± 0.82	1.0 ± 0.94	0.7 ± 1.06	n.s.
Blood vessels	3	14.6 ± 1.26	25.5 ± 1.96	3.2 ± 1.93	*C x CM. *C x NM; ***CM x NM
	7	14.9 ± 2.18	7.1 ± 1.45	2.4 ± 1.07	*C x CM. ***C x NM. *CM x NM
	14	14.8 ± 2.04	3.1 ± 1.20	2.1 ± 1.66	**C x CM. ***C x NM
	21	13.2 ± 1.55	3.1 ± 1.10	3.6 ± 1.58	***C x CM. **C x NM

Note:

* p < 0.05,

** p < 0.01,

*** p < 0.001 indicate statistically significant difference.

C = control / CM = induced movement / NM = induced movement+nicotine / NS = non-significant.

**Table 2:** Means, standard deviations and p values, according to groups and days

Variable	Day	CM	NM	Inter-group differences
		Mean + SD	Mean + SD	(p)
% Mature collagen	3	10.5 ± 2.84	5.8 ± 2.66	*CM x NM
	7	38.3 ± 4.30	7.8 ± 2.15	***CM x NM
	14	100 ± 0	9 ± 2.98	***CM x NM
	21	100 ± 0	11 ± 2.98	***CM x NM
% Immature collagen	3	89.5 ± 2.84	94.2 ± 2.66	*CM x NM
	7	61.7 ± 4.30	92.2 ± 2.15	***CM x NM
	14	0 ± 0	91 ± 2.98	***CM x NM
	21	0 ± 0	89 ± 2.98	***CM x NM

Note:

* p < 0.05,

** p < 0.01,

*** p < 0.001 indicate statistically significant difference.

CM = induced movement/ NM = induced movement+nicotine / NS = non-significant.

When groups C and CM were compared, there was an increase in the number of blood vessels on the third day after force application (p < 0.05), with a decrease after days seven (p < 0.05), 14 (p < 0.01) and 21 (p < 0.001) (Table 1). There was a greater number of osteoclast-like cells and Howship's lacunae in group CM when compared to the C group on days three (p < 0.001), seven (p < 0.001) and 14 (p < 0.01).

Fewer blood vessels were observed in all periods analyzed in group NM in comparison to group C (Table 1). Osteoclast-like cells and Howship's lacunae were reduced on the third day after the application of force (p < 0.05).

The quantity of blood vessels decreased in group NM after days three (p < 0.001) and seven (p < 0.05) when compared to group CM. Group NM had lower levels of osteoclast-like cells and Howship's lacunae, with a statistically significant difference on days seven and 14 (Table 1) in relation to group CM.

Immature collagen increased in group NM in comparison to group CM in all periods analyzed, with a statistically significant difference (Table 2).

## DISCUSSION

Nicotine is a major cytotoxic and vasoactive substance present in tobacco which causes peripheral vasoconstriction, tissue ischemia and decreased oxygen tension by reducing the infusion of oxygen to tissue. Furthermore, this substance decreases osteoblastic activity, revascularization and bone healing.^5,20^

In group NM, there were lower numbers of blood vessels when compared to group C for all periods analyzed (Table 1). When comparing groups NM x CM, we observed fewer blood vessels in group NM on days three (p < 0.001) and seven (p < 0.05), with a statistically significant difference (Table 1).

Pinto et al^3^ reported that nicotine delays angiogenesis, and as a consequence, delays the organization of connective tissue and osteogenesis. Saldanha et al^20^ demonstrated that administration of nicotine to jaw bone defects in dogs changes the density of newly formed bone tissue due to the inhibition of revascularization. Adversely, Zheng et al^4^ concluded that nicotine exposure enhances angiogenesis in rabbit model, but cannot compensate the adverse effect of vasoconstriction.

There are controversies regarding the effect of nicotine on osteoclasts. Heremyre et al^21^ observed that nicotine stimulates osteoclast-like cell differentiation in cell cultures derived from pigs; Tanaka et al^22^ also observed increased formation of osteoclast-like cells *in vitro*. We observed that nicotine reduced the expression of osteoclast-like cells and Howship's lacunae in group NM (Table 1). This result is in accordance with the findings by Yuhara et al^23^ who demonstrated that nicotine inhibits differentiation and activation of osteoclast-like cells and regulates bone metabolism in rats. Adler et al^24^ concluded that nicotine did not stimulate the formation of osteoclasts in bone marrow at doses of 1 or 2 mg / kg in rats.

In this study, we evaluated structural changes in the newly developed bone matrix when nicotine and orthodontic force were applied simultaneously. The picrosirius-polarization method allows the detection of mature and immature collagen and correlates the three-dimensional distribution of collagen fibers with the stage of bone formation.^19^ The collagen color and birefringence vary according to polymerization degree, which reflects fibers' age and diameter. First, the collagen is deposited in the form of thin fibrils that aggregate to form larger fibers or bundles.^25^

The organic matrix of alveolar bone is composed fundamentally of type I collagen (95%), proteoglycans and glycoproteins.^26^ Bone formation results from complex and inter-dependent processes, which involve osteoblast differentiation from primitive mesenchymal cells, organic matrix synthesis and maturation, until complete mineralization.^27^

The process of bone formation is associated with the formation of new capillaries from existing blood vessels.^28^ Orthodontic movement results in a rapid formation of immature bone, and later, the bone is remodeled.^29^

Immature collagen percentage in group NM increased in all analyzed periods. On day 21, there were still immature fibers, although they were thicker and more parallel. Nicotine delayed the collagen maturation process in the developed bone matrix. It was not possible to assess whether nicotine was able to inhibit the synthesis of collagen, although Theiss et al^2^ showed that during bone healing in rabbits subjected to nicotine, there were lower levels of type I and II collagen mRNA.

## CONCLUSION

In conclusion, nicotine affects bone remodeling mechanism during orthodontic movement, reducing angiogenesis, osteoclast-like cells and Howship's lacunae, thereby, delaying the collagen maturation process in developed bone matrix.
